# Weeping in silence: community experiences of stillbirths in rural eastern Uganda

**DOI:** 10.3402/gha.v8.24011

**Published:** 2015-03-31

**Authors:** Juliet Kiguli, Sarah Namusoko, Kate Kerber, Stefan Peterson, Peter Waiswa

**Affiliations:** 1 Department of Health Policy, Planning and Management, School of Public Health, Makerere University, Kampala, Uganda; 2 Saving Newborn Lives program, Save the Children, Cape Town, South Africa; 3 Global Health, Department of Public Health Sciences, Karolinska Institutet, Stockholm, Sweden; 4 International Maternal and Child Health Unit, Department of Women's and Children's Health, Uppsala University, Uppsala, Sweden

**Keywords:** stillbirth, pregnancy loss, Uganda, maternal health, postpartum depression, traditional birth attendants

## Abstract

**Background:**

Stillbirths do not register amongst national or global public health priorities, despite large numbers and known solutions. Although not accounted in statistics – these deaths count for families. Part of this disconnect is that very little is known about the lived experiences and perceptions of those experiencing this neglected problem.

**Objective:**

This study aimed to explore local definitions and perceived causes of stillbirths as well as coping mechanisms used by families affected by stillbirth in rural eastern Uganda.

**Design:**

A total of 29 in-depth interviews were conducted with women who had a stillbirth (14), men whose wives experienced a stillbirth (6), grandmothers (4), grandfathers (1), and traditional birth attendants (TBAs) (4). Participants were purposively recruited from the hospital maternity ward register, with additional recruitment done through community leaders and other participants. Data were analysed using content analysis.

**Results:**

Women and families affected by stillbirth report pregnancy loss as a common occurrence. Definitions and causes of stillbirth included the biomedical, societal, and spiritual. Disclosure of stillbirth varies with women who experience consecutive or multiple losses, subject to potential exclusion from the community and even the family. Methods for coping with stillbirth were varied and personal. Ritual burial practices were common, yet silent and mainly left to women, as opposed to public mourning for older children. There were no formal health system mechanisms to support or care for families affected by stillbirths.

**Conclusion:**

In a setting with strong collective ties, stillbirths are a burden borne by the affected family, and often just by the mother, rather than the community as a whole. Strategies are needed to address preventable stillbirths as well as to follow up with supportive services for those affected.

Whereas progress in maternal and child mortality rates is tracked in the Millennium Development Goals, stillbirths have not received similar attention (1, 2). This is despite the fact that each year an estimated 2.6 million stillbirths occur globally (3), in addition to the 2.9 million newborn deaths (4) and 289,000 maternal deaths (5).

Of the stillbirths, 1.2 million occur during labour, with the causes and solutions linked to care for mothers and newborns. Maternal conditions associated with stillbirth in low- and middle-income settings include hypertension, diabetes, maternal infection (e.g. syphilis, malaria, HIV), maternal undernutrition, obesity, and smoking (6). An estimated 40,000 stillbirths occur each year in Uganda, making it the country with the 10th highest number of stillbirths in the world. However, stillbirths are often unreported in official statistics and are invisible to health policy makers. Even in high-income countries, there are gaps in recognising the value of a dead baby, thereby dismissing the grief of the family (7). These deaths are not invisible to families – but social stigma around stillbirths and a lack of awareness about both the causes and potential solutions contribute to the silence (7).

During the design phase of Uganda Newborn Study (UNEST) in rural eastern Uganda, which aimed at strengthening health systems in order to reduce neonatal deaths, high rates of stillbirths were observed in the study area with few supporting services (8, 9). We therefore set out to explore community knowledge, attitudes, and practices, and coping of women and families affected by stillbirths, with the aim of this contributing towards designing effective preventive and care programmes. This paper is the seventh in a series on the impact of and findings from UNEST.

## Methods

### Setting

This study was part of the formative research for UNEST, a community-based newborn trial whose details have been published elsewhere (9, 10). The study was conducted in Iganga and Mayuge districts located in rural eastern Uganda, about 120 km east of the city of Kampala. The Basoga people, the predominant ethnic group in the area, make up about 10% of the population of Uganda, but their practices are similar to those of other Bantu ethnic groups in Uganda which comprise the majority. Eighty percent of the population are peasants and live on subsistence farming and retail trading which brings in an income of less than US$1 a day. TBAs are significant actors in the provision of care during pregnancy in the district, but the majority of births take place in health facilities. The main referral hospital delivers approximately 3,500 babies each year, of which 8% are stillbirths.

### Participants and data collection

This was a qualitative study employing in-depth interviews (IDIs) and observation in order to understand the context, lived realities, and interaction with the community in relation to stillbirths (11). There were a total of 29 study participants, including 7 men and 22 women (Table 1). The ages of the 14 women who delivered stillbirths ranged from 18 to 45 years with a mean age of 25 years. The parity range was 1–12 pregnancies. All study participants were of Basoga ethnicity. To recruit participants for the IDIs, we used the hospital maternity ward register to identify mothers of stillborn babies who had either a complete contact address for residential location and/or telephone numbers. Using the register information, we went to communities where these mothers came from. Additional recruitment was done through village community leaders and information gathered from interviewees.

**Table 1:** Number and type of IDI participants

Participants	*N*=29
Women who had at least one stillbirth	14
Men whose wives had at least one stillbirth	6
Grandmothers	4
Grandfathers	1
Traditional birth attendants	4

Interview guides were developed and pre-tested outside the study communities. IDIs were conducted in the local language of Lusoga with a note-taker present, and a Lusoga-speaking medical doctor supervised the research. Both the interviewer and note-taker were degree holders with over 5 years of experience in qualitative interview techniques. All of the interviews were conducted in Lusoga, tape-recorded, and later transcribed into English by the interviewer and the note-taker.

When interviewing participants, the interviewers used an exploratory technique and would probe to clearly understand and broaden the knowledge acquired. Interviewers used observation to document participants’ expressions and behaviours as they narrated their stories. Where relevant and feasible, participants went with interviewers to the place where the baby was buried to add context to the experience and enhance understanding. We explored what participants knew theoretically about stillbirths as well as their personal experiences of loss.

### Data analysis

Following transcription and translation into English, data were systematically coded and analysed manually by content analysis. The first three authors rigorously read the scripts independently and came up with codes, following what Granheim and Lundman refer to as condensation to unit analysis, content area (a process of shortening while still preserving the core), and meaning units (12). Thereafter, they met to compare and agree on the codes. Themes were identified and organised into meaningful categories, with frequencies and differences in responses by respondent type noted. Expressive and illuminating responses are presented as verbatim quotes.

Ethical approval was provided by the institutional review board of the Makerere University School of Public Health and Uganda National Council of Science and Technology. Given the emotional sensitivity around the topic, interviews took place no sooner than 6 weeks after the stillbirth event, and we used experienced research assistants from the same cultural group. Verbal consent was obtained from study participants given the low literacy rates.

## Results

The results of the IDIs reflect perceptions of the burden, definition, and causes of stillbirths as well as the coping mechanisms used by families to overcome their loss. Table 2 summarises the categories developed from broad themes of knowledge arising from the interviews.

**Table 2:** Community awareness, causes, perceptions, and beliefs about stillbirths

Condensed meaning unit	Code	Category	Theme
The dead baby is referred to as *ekintu*	A stillbirth is a thing, not fully human	How are stillbirths described	Definition of stillbirth
The baby is without life, does not breathe/no breadth of life	A stillbirth shows no sign of life	How stillbirths are understood medically	
The baby has no movements inside the mother's womb			
The baby has a soft head	Physical descriptions of stillbirths		
The baby has a long head		
The baby was disfigured/not well formed		
The baby comes out without the skin due to infection by the mother		
Maternal infections and diseases such as syphilis and malaria	Maternal infection	Perceived medical causes	Causes of stillbirth are varied
Baby is killed in the womb and syphilis pierces the baby			
There is *denyi*, much heat in the womb, and the uterus cannot hold			
Baby is pierced by syphilis, especially when syphilis is in advanced stage			
Tangled umbilical cord	Problems *in utero*		
Narrow birth canal			
Weak uterus			
Too young/women's bodies are not yet ready to hold a pregnancy	Age of mother		
Too old/some mothers feel tired after many births			
Sex during the pregnancy makes the baby come out before the time for birth	Activity while pregnant		
Falling while pregnant			
Heavy workload			
Intention to abort	Induced abortion		
Being poor and not able to afford to go for care	Poverty	Perceived societal causes	
Being kicked in the stomach	Domestic violence		
Bewitching by co-wife	Witchcraft	Spiritual causes	
Curses			
Attend antenatal care and go to hospital early	Changes in care-seeking activities	Solutions exist	
Attend a family planning clinic and space children			
Saving money; having good financial status	Changes at and around home		
Not doing heavy work and carrying heavy things			
Avoiding the use of certain herbs			
Using herbs such as *mubwa* to drink and others to rub on the stomach			
Witchcraft to block curses	Spiritual activities		
Religion and seeing a diviner			
Avoid telling other people of the pregnancy to as they may bewitch you			

### Perceived magnitude of the burden

Some participants reported that stillbirths were a common occurrence. Mothers who had experienced a stillbirth were likely to know about other women in their family or community who were similarly affected. However, it was thought that community members who were not affected by a stillbirth in their immediate family might not be aware of specific cases in the village:

We have so far had three stillbirths in this village and mine is the fourth. (Mother of a stillborn baby)

Since it is ‘*empuna*’ [*a thing*] which has not cried, they sometimes just announce to the immediate neighbours only. The rest of the village doesn't get to know about this curse. (TBA)

### Defining ‘stillbirth’

A stillbirth was locally referred to as *empuna* or *ekintu*, meaning ‘a thing’. Several definitions were used to describe stillbirths. The majority of women interviewed defined a stillbirth as a baby born dead, with no breath of life and no movement. Others described physical characteristics, such as a deformed baby with skin peeled off. A few could not define a stillbirth, even some of the women who had experienced it directly:

What we know is that a baby comes out dead, doesn't cry, and doesn't breathe. That shows us that the baby is a stillbirth. (Father of a stillborn baby)

The head was long, as if they had added another head, it was so soft and the baby was disfigured and not well formed. (Mother of a stillborn baby)

The difference between antepartum, or macerated stillbirths, and intrapartum, or fresh stillbirths, appeared to be important. Women were said to ‘weep in silence’, particularly if their baby appeared macerated or deformed. In these cases, few members of the family would be informed. Women who described this silence longed to speak about their experiences, but there was more stigma attached to these deaths than any other. The fathers of stillbirths reportedly did not cry, but expressed sadness for themselves and their wife.

### Medical and underlying causes of stillbirth

Causes of stillbirth were described firstly mainly in biomedical terms. These included maternal infections such as syphilis and malaria during pregnancy. Although syphilis was the most commonly mentioned condition, other reported physical causes of stillbirths included *denyi* (meaning there was ‘heat in the womb’), the baby becoming tired, the mother having a narrow birth canal, tying of the cord around the baby (which led to strangling of the baby) and having a ‘weak uterus’ which could not support the growth of the foetus. Particularly with maternal infection, like syphilis, the burden and responsibility seemed to fall heavily on the mother:

We know this disease [*syphilis*] can kill the baby in the womb. What I know is that they die before coming out, when syphilis is at its advanced stage. It can even come out as a miscarriage and at times the babies come out with spots on their bodies. At the end, it also causes too much heat in the body. The babies come out when already dead because of heat. (Father of a stillborn baby)

This is the third time. The first stillbirth happened at seven months, then another one followed at the eighth month at X hospital. On this one, I travelled up to until when I delivered. Everywhere I went, they told me it was syphilis. (Mother of three stillborn babies)

*Ekintu* does not make any movements when inside the uterus and … comes out without a skin due to the infection by the mother as this is transmitted to the baby. (Grandmother of a stillborn baby)

Age, body shape, and marital status were also identified as playing a role. TBAs mentioned that young mothers tended to have stillbirths as their bodies were not fully ready and prepared for child-bearing, while others, like mothers-in-law, mentioned that old mothers also lost children due to many children and having them one year after another. Some respondents noted that having sexual intercourse during pregnancy could result in a stillbirth. Several other causes were mentioned with links to societal factors and poverty. These included domestic violence, being poor and not being able to afford care, and stress from overwork: ‘A pregnant woman loses her baby if the husband kicks her in the stomach. For the husband comes home drunk and asks for his food and she delays to bring it over’ (TBA).

It was reported that if women had intentions to abort the baby, this could also result in a stillbirth. Witchcraft was commonly emphasised by mothers, especially in relation to their co-wives in a polygamous household. Some grandmothers said they would encourage their sons to marry new young women: ‘Sometimes, when one is pregnant, she does not tell other people for fear of losing the baby. One is afraid that someone may be jealous, for if you have a co-wife she will bewitch you’ (mother of a stillborn baby).

When asked about what contributes to babies dying before birth, participants mentioned delays in accessing health services. Financial barriers and lack of knowledge about when to seek care were cited as the source of the majority of delays. Another factor was the negative attitude of health workers, who were described as rude, proud, negligent, and vulgar. The midwives were quoted as often telling mothers in labour: ‘Who told you to become pregnant? Do not disturb us.’ Some young midwives were also said to abuse mothers, especially if the mother never attended antenatal care or if she had many previous pregnancies:

The health worker was only one and mothers were many, yet some health workers had gone to rest. By the time the health worker came from where she had gone to deliver another baby, she reached me when I had finished delivering a dead baby. Even the afterbirth had come out. (Mother of a stillborn baby)

Maybe if I had gone to the hospital on Friday, maybe they would have found out the baby's poor condition and tried to save it. But there was no solution. I would think the cause was the baby being tired because I delayed. (Mother of a stillborn baby)

Participants advanced several ways in which stillbirths could have been prevented, such as attending antenatal care early, saving money for emergencies, practising family planning, and avoiding a heavy workload. Divine intervention, protective witchcraft and the use of herbs during pregnancy were also regarded as important measures: ‘If I had known about my co-wife's witchcraft, then I would have consulted witchdoctors and saved my baby. My husband would have told you more of this story if he was around’ (mother of a stillborn baby).

### Effects of stillbirths on mothers, fathers, and families

The reported effects of experiencing a stillbirth on mothers and families and ways of coping are summarised in Table 3. Mothers who had a stillbirth said they were grieved because ‘every woman's happiness is giving birth’. Motherhood, and to a lesser degree fatherhood, confers societal value and is considered to be a respectful position in society. Participants noted that a stillbirth steals happiness from a family and may cause social disintegration or separation, even if followed by a live birth. Women who had multiple or consecutive stillbirths revealed that they felt ridiculed by their in-laws and community.

**Table 3:** Effect of a stillbirth on mothers and fathers and methods of coping

Participant	Response
Affected women	Mothers felt profound grief which increased with more than one stillbirth or pregnancy loss Women with consecutive stillbirths were considered cursed and lost the respect of their families and the community If it was known widely that a woman had a stillbirth, her worth may be questioned and her contributions in village meetings may not be taken seriously The woman is condemned and isolated, especially by in-laws, who encourage their son to remarry
Affected men	Experienced grief but did not cry; unlikely to mourn publicly Supportive to wives, especially in the case of a single stillbirth Blamed wife in the case of multiple stillbirths, although one man who had experienced multiple losses reported supporting his wife and not giving in to family pressure to remarry
Coping mechanisms	Support from the immediate family Older women in the extended family Trying to conceive again Counselling from sympathetic health workers Religion, belief in God

Men reported that they felt the loss strongly but reacted differently to women. Their reaction also differed between single and multiple stillbirth experiences. If consecutive stillbirths occurred, husbands were reported to reject and separate from their wives. The situation was worsened by pressure from the husband's family, who would encourage their son to leave his wife:

The men feel [*the pain of one stillbirth*], maybe [*the wife*] has some demons at her place of birth; two, maybe she is a woman with bad luck …. It can cause divorce, especially if the mother has had stillbirths twice yes … it can cause breakage of marriage and also anxiety. (Father of a stillborn baby)

However, dissolution of marriage following multiple stillbirths was not inevitable. Pressure from the community and family was further determined by the kind of relationship shared between husband and wife. It was argued that if the relationship was very good, it would not matter whether a woman had more than one stillbirth or whether the man's family had pressed him to get a divorce.

It was revealed that some family members were supportive to the mother while others would advocate for separation, in most cases regarding the woman as either a curse or a detraction to the man. Out of the 14 women who delivered stillbirths, half reported that family members were supportive; for the other half, their in-laws and co-wives were discouraging. In one report the condemnation extended to in-laws, who said the bereaved mother was filling the clan land with small graves:

Now my co-wife just tells me that ‘this time if you get pregnant again … You will be a woman.’ Otherwise she will say ‘Let her stop giving birth, the woman keeps burying every other time, she puts the family at a loss’. (Mother of a stillborn baby)

The reaction amongst those who knew about a stillbirth in the community was similar to the families: understanding and supportive if it was the first and only time a woman was having a stillbirth, but if a woman experienced more than one stillbirth, it was considered a bad omen. Such a woman's contributions in village meetings would not be taken seriously, and other women might be more likely to question her worth.

### Coping mechanisms and support structures

Regardless of whether a woman experienced one or multiple stillbirths, support from her husband and extended family was thought to be critical to recovery. Participants reported that if a woman was supported by her husband, she became stronger quickly and even conceived again quickly. Older women helped with household chores so the mothers could recover. Mothers revealed that believing in God and comfort and counselling from sympathetic health workers were sources of strength. However, participants reported that mothers of stillbirths were encouraged by families and health workers to conceive immediately to help them cope with the loss, which was not always welcome advice. Some reported the fact that they themselves survived death helped them to cope with the grief and pain, especially in the case of those with other children at home.

### Practices that affect stillbirth visibility as a community and public health concern

Study participants reported that compared to other deaths in the community, even the death of a child that lived for a short time, stillbirths are not given a proper burial, and that mock graves are used to taunt families rather than honour the baby's memory. It was frequently stated that the baby who did not cry cannot be buried in the same graveyard with adults or babies that cried, because it cannot communicate while in the spirit world. Burial practices for stillbirths ranged from clan to clan and rituals carried out when a stillbirth was brought home differed. However, it was reported that most religions did not acknowledge unbaptised or uninitiated babies (including stillbirths and newborn deaths), which limited formal application of funeral rituals. A baby would be named or given a name at a special ceremony if born alive, but a dead baby would not undergo this ritual. Other differences highlighted include digging the grave using a stick instead of a hoe if the baby did not cry. In some clans, the stillbirth was reported to be buried in a hole dug on the veranda where water flows down from iron sheets, to level the grave with the ground to make it unnoticeable. Burial of a stillborn baby was reported to take place quickly and was more often conducted by women only, although in some cases men and other community members took responsibility:

When a pregnant woman loses a baby, she is left at home, and people nearby quickly gather and bury the dead baby. It is buried in the family compound. A grave is dug using a stick. No one wails as they do for an older child, but they feel sorry for the mother as she stays home without saying goodbye to her dead baby. (TBA)

It was reported that stillbirths are not announced in the village, and in most cases people are not widely informed. However, affected women do mark the event. After a stillbirth, it was reported that a mother may not bathe for many days and will use traditional herbs to cleanse her household. In some clans the mother of a stillborn baby was prohibited from going back to her parent's home for a long period, and she was not supposed to use a type of wood, *nkandwa*, for cooking. If a mother was to go to her parents she was supposed to throw a certain herb in the fire before setting foot in the house.

## Discussion

These interviews reveal that definitions, causes of death, and solutions have biomedical and spiritual dimensions. Although community members are aware that pregnancy loss occurs, a stillbirth is primarily considered an issue affecting the woman who has directly experienced the loss. Women and families in this context have limited options for support within existing structures.

One could not miss the repetitive use of the words *empuna* and *ekintu*, both referring to ‘a thing’ in reference to stillbirths. Similar to the use of the English word stillborn as a synonym for useless or ineffectual (7), it is unsurprising that the local term makes no reference to the life that has been lost. Defining the stillbirth as a thing that does not cry provoked different burial practices. In Tanzania, the differences in burial practices for ‘immature’ babies (most stillbirths) are due to the fact that they are not considered fully human and therefore potentially threatening (13).

We found that participants first described stillbirth to be medically related, followed by societal and spiritual causes. Syphilis in particular was described as a major factor, even though it is less likely to be a cause of stillbirth than malaria in this setting (6). Malaria was not commonly mentioned as an associated cause. Notably, domestic violence was raised as an important contributor to stillbirths and was reportedly widespread. One study in rural western Uganda found that up to 70% of men and 90% of women believed that violence against a female partner was justifiable in certain circumstances (14).

The issues that lead to delays in care-seeking and receiving care are well known in the literature (15, 16), but we were surprised at the level of knowledge in this community. A possible explanation could be learning from the many sessions women attend during pregnancy and the search for an explanation for their own loss. While unknown causes were associated with superstition, there was no indication that participants thought these stillbirths were inevitable, a finding seen in other African settings (17). The fear of being accused of having an induced abortion has been reported by women in other settings (13), but respondents in this study were concerned that even an intention to terminate the pregnancy could cause a stillbirth.

It is culturally assumed that a community will not grieve the loss of ‘a thing’. The burial of a stillborn baby is done in private, mainly by women, with little opportunity for public mourning or condolences. Women who experienced antepartum stillbirths in particular reflected the need to be ‘weeping in silence’ to avoid further stigmatisation for themselves and their families. Respondents did not mention peer support or seeking out other women who had experienced stillbirth as a mechanism to cope with the loss, although older women were mentioned as a source of physical help with chores and household responsibilities.

These findings reaffirm that despite the magnitude of stillbirths and the intersection with maternal and child health, the health system and community support structures have been largely unresponsive both in terms of prevention efforts and providing support to affected families (7, 13). To avoid blame and potential divorce, the couple was often counselled, even by health workers, to have another baby to ‘replace’ the baby who was stillborn, despite risks of conceiving quickly after loss (18). In addition to efforts to prevent stillbirths within the context of maternal and newborn care, there is a need for education to lessen the stigma and create supportive spaces for families to grieve. Experiencing a stillbirth is not an acute event; literature from high-income countries demonstrates that the trauma of stillbirths may remain up to 18 years after the loss (19). Such efforts include incorporating bereavement support in health worker training as well as engaging community structures, and sensitising communities to the problem beyond the individual woman or family (7).

A limitation of our study is the small number of women interviewed who had experienced a stillbirth, only one of whom had delivered outside a health facility. Mothers who delivered stillborn babies at home without accessing the healthcare system, or those experiencing additional barriers to disclosing stillbirths, were difficult to locate and their views may be under-represented. However, we triangulated data across interviews with mothers who experienced stillbirth, and interviews with other key actors who could speak to the experience of home deliveries and social norms, such as TBAs.

## Conclusion

In Uganda, as elsewhere, stillbirths represent a massive loss in terms of mortality but also an additional emotional burden to be borne by women. Stillbirth is also felt by the father and immediate family members, but in most cases it is not considered a community concern or a priority issue for health programming. The capacity of the health system needs to be built not only to account for and prevent stillbirths but also to follow up and provide physical, psychological, and emotional support to women and families who have experienced loss.
